# Blood Flow Forces in Shaping the Vascular System: A Focus on Endothelial Cell Behavior

**DOI:** 10.3389/fphys.2020.00552

**Published:** 2020-06-05

**Authors:** Pedro Campinho, Andrej Vilfan, Julien Vermot

**Affiliations:** ^1^Institut de Génétique et de Biologie Moléculaire et Cellulaire, Illkirch, France; ^2^Centre National de la Recherche Scientifique, UMR 7104, Illkirch, France; ^3^Institut National de la Santé et de la Recherche Médicale, U964, Illkirch, France; ^4^Department of Development and Stem Cells, Université de Strasbourg, Illkirch, France; ^5^Department of Living Matter Physics, Max Planck Institute for Dynamics and Self-Organization, Göttingen, Germany; ^6^Department of Condensed Matter Physics, J. Stefan Institute, Ljubljana, Slovenia; ^7^Department of Bioengineering, Imperial College London, London, United Kingdom

**Keywords:** cardiovascular, stretch, low Reynolds number, angiogenesis, *Danio rerio* (zebrafish), live imaging, cilia, mechanotransduction

## Abstract

The endothelium is the cell monolayer that lines the interior of the blood vessels separating the vessel lumen where blood circulates, from the surrounding tissues. During embryonic development, endothelial cells (ECs) must ensure that a tight barrier function is maintained whilst dynamically adapting to the growing vascular tree that is being formed and remodeled. Blood circulation generates mechanical forces, such as shear stress and circumferential stretch that are directly acting on the endothelium. ECs actively respond to flow-derived mechanical cues by becoming polarized, migrating and changing neighbors, undergoing shape changes, proliferating or even leaving the tissue and changing identity. It is now accepted that coordinated changes at the single cell level drive fundamental processes governing vascular network morphogenesis such as angiogenic sprouting, network pruning, lumen formation, regulation of vessel caliber and stability or cell fate transitions. Here we summarize the cell biology and mechanics of ECs in response to flow-derived forces, discuss the latest advances made at the single cell level with particular emphasis on *in vivo* studies and highlight potential implications for vascular pathologies.

## Introduction

The endothelium is a squamous cell monolayer that lines the lumen of all blood vessels and keeps the vessel interior sealed from the neighboring environment. Endothelial cells (ECs) are interconnected by cellular junctions that confer selective permeability and have their apical side facing the vessel lumen where fluids, nutrients, gases, cells, hormones, and other factors circulate to reach the entire organism. To ensure distribution to virtually all tissues in the body during embryonic development, ECs assemble into a vast tree-like network of tubes – the vascular system. Network development is achieved in a series of stereotyped steps. First, a primary network that consists mostly of the main axial vessels, the aortic arches, and the umbilical vessels is formed in a process termed vasculogenesis (Downs et al., 1998; Swift and Weinstein, 2009; Potente et al., 2011; Arora and Papaioannou, 2012; Frisdal and Trainor, 2014). In zebrafish, EC precursors – the angioblasts – migrate from both sides of the lateral plate mesoderm to meet at the embryonic midline where they coalesce into a chord like structure that later splits into two axial vessels in which eventually a lumen opens up to form the main artery and vein before circulation initiates (Swift and Weinstein, 2009; Sato, 2013). In amniotes, the process is slightly different and first two independent lateral dorsal aortae are formed at each side of the notochord that later fuse to give rise to the common dorsal aorta (Strilic et al., 2009; Sato, 2013). The rest of the vasculature (secondary network) arises in the presence of blood flow and is constantly being remodeled to adapt to embryonic growth and to new physiological demands like the irrigation of newly formed organs. This process is called angiogenesis and will be the main focus of this review. New branches arise from pre-existing vessels in a process called sprouting angiogenesis that involves the differentiation of a tip cell leading the way as well as stalk cells that follow, although these roles are not fixed and cells can dynamically swap positions (Geudens and Gerhardt, 2011; Siekmann et al., 2013). Afterward, the newly growing sprouts fuse with one another or with previously existing vessels, thus forming new connections in a process named anastomosis (Betz et al., 2016). Alongside, the newly formed connections become patent, allowing the formation of a lumen where blood can circulate. Finally, the network is optimized by the stabilization of some branches while others regress in what is known as vascular pruning (Betz et al., 2016). Another important type of angiogenesis involved in network remodeling and optimization is the so-called intussusceptive (splitting) angiogenesis in which ECs from opposing vascular walls protrude inwards, toward the vessel lumen, forming transluminal pillars that can ultimately split a pre-existing vessel in two (Makanya et al., 2009).

**FIGURE 1:**
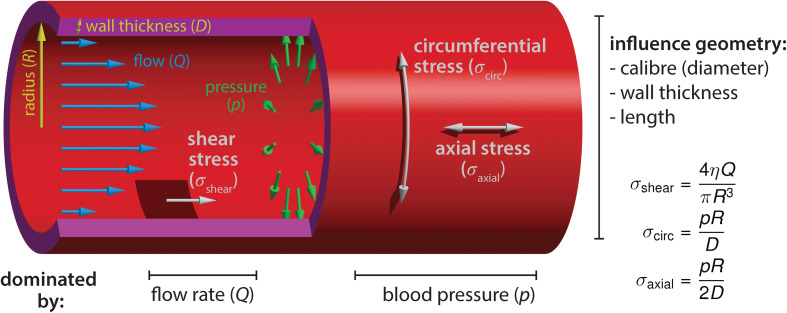
Blood flow-derived forces. Schematic representation of the mechanical forces experienced by endothelial cells due to blood circulation inside the vessels. The blood flow, measured by the volume flow rate *Q*, causes a shear stress σ_shear_ on the wall. The shear stress depends on the flow rate *Q*, the blood viscosity η and the vessel radius *R*. The relationship shown is valid in laminar flow, occurring at low Reynolds numbers. Independently of the flow, the hydrostatic pressure *p* causes a circumferential (hoop) stress σ_circ_ and an axial stress σ_axial_. Contrary to shear stress, they increase linearly with the vessel radius. The circumferential stress has twice the magnitude of the axial stress. The equations hold for thin walls (*D*≪*R*). Furthermore, we assumed that no external forces are acting on the vessel, such that the pressure forces are fully counterbalanced by the stress in the walls (depending on the mechanical environment, the force-free condition may not always be fulfiled, especially in the axial direction).

The majority of growth and remodeling of the vascular network takes place when blood circulation has already initiated and the endothelium is exposed to flow-derived mechanical forces such as shear stress, circumferential stress and axial stress (Figure 1). Shear stress is the force parallel to the tissue surface that arises due to shear flow of the viscous fluid and depends on the flow rate, viscosity of the blood, as well as on the geometry of the tube. The other two forces are governed by the intraluminal pressure. Circumferential stress is the force tangential to the vessel wall in the azimuthal direction (around the circumference) and axial stress is the force along the longitudinal (long) axis of the vessel. These three stresses dictate blood vessel mechanics and influence geometrical parameters of vessels, such as the radius, wall thickness, and length (Hoefer et al., 2013). Although the importance of axial stress has long been recognized, its impact on blood vessel morphogenesis is still less well studied (Humphrey et al., 2009). EC behaviors induced by shear stress or circumferential stretch are better studied, particularly during embryonic development, and will be the focus of our review. Besides intravascular flow, we will also mention interstitial (transvascular) flow due to vessel permeability, which generates shear stress that has been shown to influence sprouting angiogenesis and is particularly relevant in the context of tumor vascular biology (Kutys and Chen, 2016).

As a physical stimulus, blood flow can be highly diverse and since ECs are sensitive to many different features of flows it is important to consider the details of the flow profiles in the developing vascular system. As the heart is maturing during the earliest steps of vascular morphogenesis, the flow generated as a result of its immature activity is constantly changing (Boselli et al., 2015). As a consequence, different types of flow can be generated in the same vessel as it matures. Embryonic blood flow falls into the low Reynolds number regime; see in zebrafish (Anton et al., 2013; Goetz et al., 2014), the early embryonic vascular network in mouse (Jones et al., 2004) and chick (Al-Roubaie et al., 2011), where viscous forces dominate and inertia is negligible (Freund et al., 2012). This leads to flow profiles that are not intuitive, from unidirectional to bidirectional flow and pulsatile to disturbed flow, all of them in theory capable of eliciting distinct cellular behaviors (Freund et al., 2012; Boselli et al., 2015) or different transcriptional responses (LaMack and Friedman, 2007; Yee et al., 2008; Feaver et al., 2013). The most extreme flow rates are certainly experienced by the ECs of the heart outflow tract (Duchemin et al., 2019a) as well as endocardial cells, where the flow profiles can be mapped and the shear stress dissected into components (Boselli et al., 2017), all of which may contain mechanical information that is sensed by ECs. It has been proposed that the difference between arterial and venous identity is enhanced by flow (Le Noble et al., 2004) but the complexity is augmented by the cell behaviors involved in the process which will be discussed here. In addition, the mechanics of the vascular system follows basic principles of blood vessel mechanics described in adults. For example, the maturing main trunk artery in the zebrafish embryo deforms at each pulse of heart contraction (Anton et al., 2013; Campinho et al., 2018). Overall, significant advances coming from progresses in live-imaging performed in zebrafish and other species in combination with tailor-made sophisticated image analysis algorithms and mathematical modeling will be discussed here in the perspective of cell behaviors.

Flow-derived mechanical cues (Jones et al., 2006; Freund et al., 2012; Duchemin et al., 2019b) in combination with a tightly regulated genetic and metabolic program (Potente and Makinen, 2017) are well known to be essential for controlling the growth, identity and shaping of the vascular network. Tissue morphogenesis and homeostasis is driven by coordinated changes at the single cell level, such as cell shape changes, rearrangements, proliferation and extrusion or cell fate specification, which are well described for epithelial morphogenesis (Guillot and Lecuit, 2013). Recent studies have shed light on cellular changes triggered by blood flow in the developing vasculature. In this review, we attempt to summarize the latest advances on flow-induced single EC behavior in the early development of the vascular system *in vivo*.

## Endothelial Cell Polarization and Migration

Flow induced polarization of cells within the plane of the endothelium is well established and certainly participates to the morphogenetic changes activated by flow forces. Cultured ECs respond to shear stress by becoming elongated and oriented along the axis of flow (Levesque and Nerem, 1985) via re-organization of the cytoskeleton (Wojciak-Stothard and Ridley, 2003; Noria et al., 2004). Shear stress sensing can occur at junctional complexes, membrane receptors or channels and at the glycocalyx – for review see Duchemin et al. (2019b), Shurer et al. (2019). The glycocalyx is a thin sugar matrix covering the apical side of ECs present at blood flow onset that is required for the formation of a normal vascular network in chicken (Henderson-Toth et al., 2012). Moreover, its selective stimulation results in endothelial nitric oxide production, a potent vascular signaling molecule (Bartosch et al., 2017). Shear stress induced cytoskeletal remodeling works via force transmission at the cellular junctions (Baeyens et al., 2016a; Duchemin et al., 2019b), which simultaneously causes tension to increase at junctional platelet endothelial cell adhesion molecule (PECAM)-1 while decreasing at junctional vascular endothelial (VE)-cadherin. This is in line with observations that even acute loss of blood flow does not lead to clear changes in the tension at the junctional VE-cadherin in the zebrafish dorsal aorta (DA) (Lagendijk et al., 2017). The tension rising across junctional PECAM-1 promotes its association with vimentin that in turn transmits back myosin-generated forces to PECAM-1, thus working as a signal amplification mechanism. Tension building-up across the cytoskeleton appears to be counterbalanced by phosphoinositide (PI) 3-kinase dependent inhibition of actomyosin contractility, which prevents ECs from becoming overstretched and facilitates cellular rearrangements (Angulo-Urarte et al., 2018). On the other hand, ECs exposed to cyclic mechanical strain re-orient perpendicularly to the axis of strain by remodeling their cytoskeleton (Thodeti et al., 2009). Here the force sensing mechanism works via the transient receptor potential channel 4, which results in β1-integrin activation at focal adhesions through PI3-kinase activity. Recently, β1-integrin has also been proposed to function as a sensor of the flow direction (Xanthis et al., 2019) and as a regulator of *klf2a* (Krüppel-like factor 2, *klf2*) expression in zebrafish (Renz et al., 2015). At the tissue scale, the endothelium mechanical state is thus defined by the influence of forces generated both by the mechanical environment (flow, stretch) and the EC contractile activity.

**FIGURE 2:**
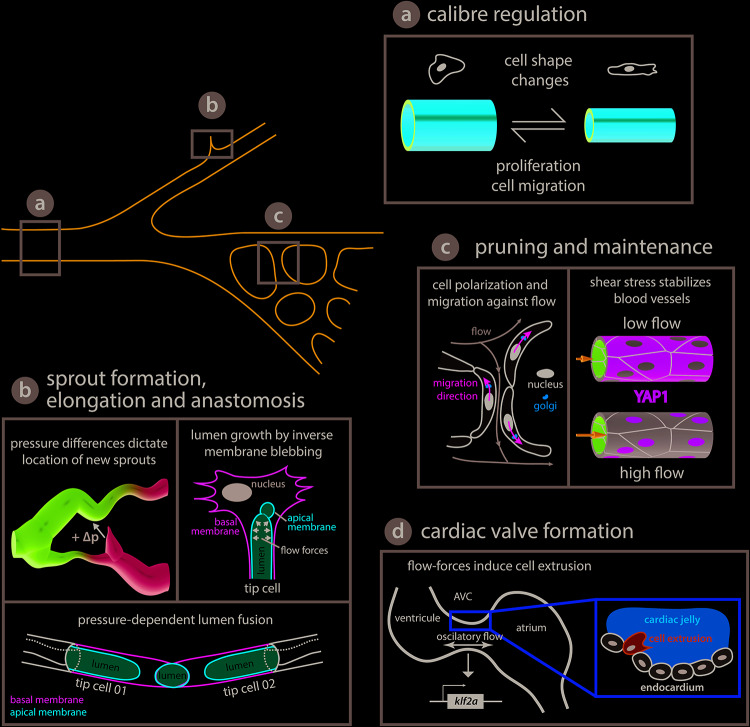
Endothelial cell behaviors triggered by flow-derived mechanical cues during vascular network growth and development. **(a)** Arteries adjust their caliber in response to hemodynamic forces. Arteries can either reduce their diameter via coordinated cell shape changes (Sugden et al., 2017) or increase the diameter by cell proliferation and migration toward enlarging vessels (Udan et al., 2013; Poduri et al., 2017). **(b)** Blood pressure has been shown to play a central role in network growth. The location and growth of newly forming sprouts is set by local differences in blood pressure (high and low pressure, magenta and cyan, respectively, Ghaffari et al., 2015). In addition, intraluminal pressure causes inverse blebbing of the apical membrane that drives lumen growth in new sprouts – drawn after Gebala et al. (2016). Similarly, during vessel anastomosis the apical membranes of two adjacent tip cells are pushed toward each other in a blood pressure-dependent fashion – illustration after Lenard et al. (2013). **(c)** Vascular networks can be remodeled in response to shear stress. Cell polarization and migration against the flow direction can lead to retraction of poorly irrigated vessels – sketched after Franco et al. (2016) – while vessels exposed to vigorous flow are kept by shear stress induced translocation of YAP to the nucleus – depiction after Nakajima et al. (2017). **(d)** Flow-derived mechanical cues can lead to cell extrusion and the formation of new structures such as the atrioventricular canal (AVC) valve or trigger cell fate transitions as seen during hematopoietic stem cell formation (see Figure 3). During AVC valve formation oscillatory blood flow triggers expression of the transcription factor *klf2a*, which is thought to promote invasion of the cardiac jelly by endocardial cells thus initiating the formation of the valve leaflets. Diagram after Heckel et al. (2015) and Steed et al. (2016).

Besides altering their shape and orientation, ECs can also migrate in response to flow forces. A recent *in vitro* study reported EC migration in response to gradients of shear stress created by impinging flow (Ostrowski et al., 2014). However, the migration direction varied depending on the cell density; high confluent cells migrate against the flow direction whereas low-density (isolated) cells migrate with the flow. In addition, cell orientation relative to flow seems to be cell type dependent. Aortic valve ECs elongate perpendicularly to the unidirectional shear stress generated by steady laminar flow (Butcher et al., 2004), contrary to ECs that align with the shear axis (Levesque and Nerem, 1985). Similarly, endocardial cells tend to converge toward the area of high shear and oscillatory flow and do not exactly follow the direction of the net flow during the initial steps of valve development in zebrafish (Boselli et al., 2017). Despite the crucial role for flow-induced cell polarization and migration during development and remodeling of the vascular network, the cellular and molecular mechanisms involved have remained elusive until recently. Besides the macroscopic changes in cell shape and alignment, cell polarization is also reflected by the internal organization of cellular organelles or compartments which is particularly relevant in migrating cells or in establishing tissue polarity (Figure 2). Two independent studies used mouse retinas and zebrafish embryos to follow EC polarity *in vivo* by monitoring the position of the Golgi apparatus relative to the cell nucleus (Franco et al., 2016; Kwon et al., 2016). This approach revealed that ECs polarize against the flow direction with the Golgi located in front of the nucleus and that the degree of polarization is positively correlated with the flow and shear stress magnitude (Franco et al., 2016). The response is similar in other developing blood vessels and is reversible depending on the flow forces ECs experience (Kwon et al., 2016). Mechanistically, the Golgi complex localization at the leading edge of migrating ECs requires the Apelin receptor, a G-protein coupled receptor. Nevertheless, EC response to flow differs between arteries and veins, with arterial ECs displaying, in general, a higher degree of polarization (Kwon et al., 2016). In addition, shear stress-induced EC polarization and migration requires Dachshund homolog 1 DACH1 dependent activation of the C-X-C motif chemokine (CXC) ligand 12 and its CXC receptor 4 (Chang et al., 2017). The signaling axis formed by the CXC receptor 4 – ligand 12 is known to be involved in cell migration against blood flow during artery formation (Xu et al., 2014), which is coupled to sprouting angiogenesis by Notch function and its ligand Delta-like 4 (Pitulescu et al., 2017). The transcription factor DACH1 is strongly expressed in developing arteries exposed to low (variable) flow and down-regulated in mature vessels exposed to high (laminar uniform) blood flow. Thus, DACH1 levels of expression depend on blood flow intensity (Chang et al., 2017). Considering that ECs experience a broad range of mechanical stimuli, including different flow profiles, numerous evidences indicate that their sensitivity is tuned to certain differences in the flow patterns. They may include shear stress magnitude, direction, temporal gradients and frequency content (Orr et al., 2007; Simmers et al., 2007; Feaver et al., 2010, 2013). When simply considering shear, cultivated ECs display preferential response to a range of shear stress which is cell line dependent (Baeyens et al., 2015). Where is this selectivity to certain flow features coming from? This may be partly explained by cellular context. For example, EC response is modulated by Vascular endothelial growth factor receptor (VEGFR) 3 expression levels, which tune the sensitivity of the shear stress sensor PECAM-1/VE-cadherin. Furthermore, non-canonical Wingless-related integration site (Wnt) signaling provides an additional layer of modulation of EC sensitivity to flow (Franco et al., 2015). Genetic knock-out of Wnt5a and Wnt11 produced mouse embryos with ECs that became responsive to lower shear stress levels. Wnt5 and Wnt11 depleted ECs have no alteration in the expression of many flow responsive genes, suggesting that Wnt acts downstream of the flow sensing machinery (Franco et al., 2015). This just highlights one mode of regulation but there are many other ways, including modes where the mechanosensitive apparatus could be directly modulated to tune flow sensing.

## Sprouting Angiogenesis

Blood flow forces have been implicated in the regulation of new angiogenic sprout formation as well as elongation. Nevertheless, the flow-derived forces and the cellular responses at work are only now starting to be understood. Because they allow system simplification and control over several parameters, *in vitro* studies using microfluidic devices have contributed extensively to our understanding of sprouting regulation by blood flow. Interstitial (transvascular) flow generated between two parallel channels lined by ECs, separated by a collagen matrix, revealed that sprouting is enhanced by interstitial flow and always initiates in the channel without shear stress (Song and Munn, 2011). At the cellular level, this behavior certainly depends on a global change in gene expression profile governed by epigenetic changes modulated by active histone deacetylase-1 (HDAC1) (Bazou et al., 2016). These gene expression changes include factors involved in cell migration, such as matrix metalloproteinases (Galie et al., 2014; Bazou et al., 2016). Yet, the role of flow-derived forces might have a more global control of the network growth by dictating the location of sprout initiation in response to spatial patterns of shear stress (Galie et al., 2014). Once again, the response is dependent on the flow profile ECs experience. For example, flow-derived forces can compete to regulate angiogenic sprouting and sometimes have opposite effects on sprouting location (Akbari et al., 2019). Sprout formation is supressed by impinging flow stagnation or by laminar shear stress, at the bifurcation point or downstream of it, respectively, while combined application of transvascular and intraluminar flow promotes angiogenic sprouting (Akbari et al., 2019). Thus, flow forces are integrated both at cellular and tissue levels. As a consequence, growing tissues should be considered as more than the sum of isolated cell responses.

Sprouting angiogenesis constitutes a great model system to study collective cell behaviors elicited in remodeling tissues in response to biological flows *in vivo*. Live-imaging of the quail embryo capillary plexus combined with computational fluid dynamics allows simultaneous characterization of hemodynamics and network remodeling. Here, the key predictor for sprout location is directly associated with pressure differences (Figure 2) (Ghaffari et al., 2015). The sprouts always originate from a low pressured vessel and connect to a higher pressured vessel. In addition, sprouts start at local shear stress minima except in confluence regions where two blood streams meet. Importantly, sprouting requires a positive pressure difference between two locations and the sprout elongation rate is proportional to that difference (Ghaffari et al., 2015). These pressure differences are most likely associated with interstitial flows and it will be interesting to assess if they generate meaningful signaling molecule gradients that could explain the activation of sprouting behavior. For example, in the zebrafish branchial arches flow-induced *klf2a* expression activates endothelial-specific expression of the *mir-126* microRNA, which via inhibition of *spred1* is permissive for Vegf-driven sprouting since *spred1* normally inhibits Vegf signaling (Nicoli et al., 2010). Cilia dependent shear stress sensing appears to be involved in venous sprout formation since reduced *pkd2* expression leads to network defects that are similar to those observed in the absence of blood flow (Goetz et al., 2014). Even though numerous breakthroughs in the field have helped to better understand how flow can affect cell behaviors during angiogenic processes, we are only starting to grasp how flow-derived mechanical cues affect EC sprouting. Important future work is expected in understanding the mechanosensing involved in triggering sprouting, its downstream effectors and the cross-regulation with biochemical signals involved in this process.

## Intussusceptive Angiogenesis

Intussusceptive angiogenesis has also been shown to be governed by blood flow forces (Makanya et al., 2009). The correlation between blood flow dynamics and network optimization via intussusceptive angiogenesis was first demonstrated by locally altering the blood flow in the chick chorioallantoic membrane (CAM) and observing an accelerated formation of intussusceptive pillars (Djonov et al., 2002). Flow simulations revealed that formation of new pillars is restricted to regions of low shear stress, thus shaping the developing network (Lee et al., 2010). In addition, intussusception can also be driven by tissue-level forces. This was demonstrated by application of uniaxial stretch to the CAM, which revealed that pillar density in stretched regions of the CAM was increased relative to non-stretched control regions (Belle et al., 2014). Live-imaging of the zebrafish caudal vein (CV) plexus and hemodynamic simulations confirmed that new pillars arise at shear stress minima and the direction of elongation and pillar fusion follows shear stress patterns (Karthik et al., 2018). In addition, this work emphasized that development of the zebrafish CV plexus is achieved by the concerted action of sprouting and intussusceptive angiogenesis, thus proving to be a valuable model to study the cross-regulation between these two modes of angiogenesis *in vivo* (Karthik et al., 2018). How do ECs decide between sprouting or intussusceptive angiogenesis? Is it one followed by the other, as suggested for the developing zebrafish CV plexus (Karthik et al., 2018)? It is possible that different flow profiles trigger either sprouting or intussusception or simply that the same mechanical stimulus triggers distinct cell behaviors in different environments like the vascular bed, location within the embryo or developmental context, since these could mean that cells express different gene profiles and/or are exposed to different types of biochemical signals.

## Anastomosis and Lumen Formation/Expansion

Vessel fusion can occur between two tip cells of newly forming sprouts (head-to-head anastomosis) or between a tip cell and a perfused blood vessel (head-to-side anastomosis). In both instances, a lumen that runs inside the entire newly established connection is created, in a proximal to distal fashion, thus forming a multicellular tube were blood circulates. This process can occur either in the presence of blood flow by membrane invagination or in the absence of flow through chord hollowing (Betz et al., 2016). Here, we are going to focus on flow-dependent anastomosis and summarize the most recent findings obtained using the zebrafish and mouse models. In lumenized angiogenic sprouts the proximal apical membrane of the tip cell invaginates toward the cell center due to blood pressure inside the lumen of the adjacent stalk cell (Figure 2; Herwig et al., 2011). The apical membrane invagination is then extended until it reaches the most distal side of the tip cell where it fuses with the lumen of the connecting blood vessel or sprout. The resulting section of vessel is formed solely by one cell, which is later converted into a multicellular tube by stereotyped cellular rearrangements that involve EC splitting and junction remodeling (Herwig et al., 2011; Lenard et al., 2013).

Lumen formation and growth appear to be driven by a newly described blood pressure-dependent cellular process named inverse membrane blebbing (Figure 2), which is analogous to bleb-driven cell locomotion except that the membrane bulges in the opposite direction toward the cell body; hence the name inverse blebbing (Gebala et al., 2016). Blood pressure inside the lumen causes the apical membrane to detach from the actomyosin cortex forming blebs at locations where the membrane-cortex anchoring is weak. Following formation and expansion by continuous blood pressure, blebs can either persist causing the lumen to grow or retract by assembly and contraction of new actomyosin cortex inside the bleb. Selective bleb retraction at the sides of the expanding lumen combined with bleb retention at the tip of the expanding lumen confers directionality to the process (Gebala et al., 2016). Lumen growth via inverse membrane blebbing can occur in angiogenic sprouts that contain one or two tip cells thus leading to unicellular or multicellular lumen formation, respectively (Gebala et al., 2016). Further work will be necessary to understand how the correct location for lumen growth and expansion is sensed and restricted by ECs, so that blebs can be selectively maintained or retracted in order to steer lumen growth in the correct direction. The process appears to be driven by pressure differences, but what are the mechanosensing elements at play and how these stimuli are transduced to the cytoskeleton remains elusive. Likewise, how the transformation of a unicellular tube into a multicellular tube by EC splitting and junction remodeling is regulated at the molecular level to achieve such cellular behaviors in response to blood flow still remains an open question. Is the junction remodeling solely driven by shear stress sensed by junctional PECAM-1/VE-cadherin, which then transmits these signals down to the cytoskeleton, or is pressure also involved? It is possible that ZO1 mechanosensation plays a role as recently observed for epithelial tissues (Schwayer et al., 2019).

## Vessel Pruning and Stabilization

The formation of vascular networks, plexus in particular, is not always pre-patterned and this leads to the formation of highly branched networks that are convoluted and require optimization. This process is driven by blood flow forces and occurs through selective elimination of poorly irrigated branches (pruning) and, simultaneously, maintenance of vigorously irrigated blood vessels (stabilization). Nevertheless, the cellular behaviors and signaling pathways that are involved in vascular network optimization in response to flow have only recently begun to be understood.

Experimental manipulation of hemodynamics in the developing zebrafish demonstrated that in the brain vasculature pruning occurs by lateral migration of ECs from vessels exposed to low and variable blood flow toward the neighboring vessels with stronger flow that are kept (Chen et al., 2012). Similarly, vessel pruning associated with blood flow changes was also reported in the developing vasculature of the zebrafish eye via EC rearrangements and cell death (Kochhan et al., 2013) as well as in the subintestinal vein plexus where pruning is driven by lateral migration of ECs and involves the formation of unicellular tubes by EC self-fusion (Lenard et al., 2015). In both examples, the sequence of events leading to vessel pruning resembles vessel anastomosis in reverse order. Results obtained in the developing vasculature of the mouse retina and the zebrafish embryo confirm that pruning occurs via migration of ECs away from the network branches with low flow and toward the ones with high flow levels (Franco et al., 2016). This leads to an interesting model explaining how blood flow can participate in network optimization. The model proposes that shear stress-induced EC polarization directed against the main flow direction serves as an instructive cue defining EC migration direction (Figure 2). EC movement is usually directed toward higher levels of shear stress leading to the simultaneous retraction and elimination (pruning) of blood vessels exposed to low blood flow along with the stabilization of vessel connections experiencing higher blood flow (Franco et al., 2016). As a consequence, blood vessel maintenance (stabilization) is working hand-in-hand with vessel pruning during network optimization. This is exemplified in many remodeling vascular systems where flow limits angiogenesis to stabilize the network. For example, blood circulation downregulates the expression of the *cxc* receptor *4a*, a known proangiogenic factor (Packham et al., 2009; Bussmann et al., 2011). By modulating the expression of the *cxc* receptor *4a*, blood flow promotes the maintenance and stabilization of newly established vascular connections in the zebrafish trunk and brain vasculature. Similarly, non-canonical Wnt signaling has been proposed to tune EC response thus preventing premature vessel regression (Franco et al., 2015). Flow can also positively regulate gene expression to control vessel stability by modulating the expression of the sphingosine-1-phosphate receptor 1 (S1P1) and the transcription factor Yes-activated protein (YAP) 1 (Jung et al., 2012; Nakajima et al., 2017). The S1P1 is a G protein-coupled receptor that stabilizes the primary vascular network by inhibiting angiogenic sprouting and enhancing cell-to-cell adhesion (Gaengel et al., 2012). In cultured human ECs, nuclear translocation of YAP is regulated by shear-dependent conformational changes induced to the actin cytoskeleton that in turn affect the binding of angiomotin (AMOT) to YAP (Nakajima et al., 2017). The authors propose that under low flow conditions YAP1 is bound to AMOT and thus kept in the cytoplasm, but when blood flow is high, shear stress increases the number of F-actin bundles in the cytoplasm causing AMOT to associate with F-actin and release YAP to the nucleus (Figure 2).

It is worth mentioning that YAP and TAZ also have an important role during sprouting angiogenesis. In the mouse, genetic loss of YAP/TAZ leads to arrested sprouting, while forced expression of TAZ in the nucleus causes increased sprouting. The ECs mechanical environment is slightly different here because the vessels are not perfused, but are subjected to stretch. In response to stretch, YAP/TAZ activity increases and promotes EC proliferation and cell rearrangement by increasing VE-cadherin turnover and the formation of junction associated intermediate lamellipodia (Neto et al., 2018). Interestingly, YAP/TAZ activity is enhanced by lowering levels of Bone morphogenetic protein (BMP) signaling, thus suggesting that ECs integrate mechanical stimuli with biochemical signals in order to regulate junction remodeling and cell rearrangements necessary for tissue homeostasis maintenance (Neto et al., 2018).

The effectors of flow forces during vessel pruning and stabilization are diverse and can have opposite roles in remodeling vascular tissues. The ECs have thus important capacities of mechanical and biochemical signal integration in order to adopt the adequate cellular behavior in each context. If this process is not functioning properly will, most likely lead to pathological response.

## Vessel Caliber Regulation

Vascular network remodeling can also be achieved by altering the caliber (diameter) of the existing blood vessels and usually this takes place in response to hemodynamic changes. Work in the developing pharyngeal arch arteries of the chicken embryo combined fluid dynamics simulations with artery occlusion experiments to demonstrate that other factors besides wall shear stress, such as complex shear stress patterns or pressure, are necessary to account for the morphological changes observed (Lindsey et al., 2015). Nevertheless, a developmental atlas of the pharyngeal arch arteries morphology correlated with fluid dynamics maps suggests that shear stress is the main driver of artery growth while pressure is the secondary driver (Lindsey et al., 2018). The situation may be more complicated in the developing vascular network in the mouse yolk sac where two distinct modes of artery caliber expansion are observed in response to changes in blood flow. Fusion of two adjacent vessels in the case of strong flow conditions or by migration of ECs from neighboring capillaries to expand the larger arteries (Udan et al., 2013). The signaling pathways involved also start to be uncovered. It has been proposed that VEGFR3 modulates EC response to shear stress such that above a given threshold vessels enlarge in response to shear stress while below that threshold vessels narrow thus keeping homeostasis (Baeyens et al., 2015). Supporting this view, reduced expression of VEGFR3 in zebrafish embryos and adult mice leads to decreased artery diameter (Baeyens et al., 2015). Blood flow-driven expression of the BMP receptor, activin receptor-like kinase 1 (*Alk1*), appears to limit the caliber of nascent arteries (exposed to vigorous flow) since zebrafish mutant embryos for *alk1* display aberrantly enlarged arteries (Corti et al., 2011). This phenotype is initially due to an increase in the EC number and followed by a decrease in the EC density (cell enlargement), which eventually leads to the formation of flow-dependent arteriovenous malformations (AVMs) (Corti et al., 2011). It was proposed that the abnormal artery dilation in *alk1* mutants was due to cell migration defects (Rochon et al., 2016) as well as loss of expression of the Transforming growth factor-β receptor *endoglin* (eng) and its vasoconstrictive action (Corti et al., 2011). Accordingly, the ECs of the zebrafish DA continue to enlarge causing expansion of the artery caliber in *eng* mutants when normally, at that stage (72 h post-fertilization), it should decrease (Sugden et al., 2017). At the cellular level, EC shape changes explain the DA caliber reduction in response to blood flow as cells elongate after being exposed to flow (Figure 2). In *eng* mutants ECs fail to elongate and, moreover, mutant cells transplanted into wild type embryos caused intersegmental vessels to enlarge locally (Sugden et al., 2017). How the flow-derived cues are transduced to *eng* and what is the cellular machinery driving the cell shape changes downstream of *eng* still remains elusive. In mouse retinas, mosaic deletion of *eng* was used in combination with an excision reporter to reveal that AVMs have an arterial origin despite *Eng* mutant cells displaying a venous phenotype (Jin et al., 2017). Besides a direct flow activation, *Eng*-dependent EC response seems to be involved in ligand-mediated signaling, since the response to VEGF signaling was partially affected in *eng* loss of function (Jin et al., 2017). It is to note that a distinction between EC polarity and migration needs to be done in *Eng* mutants. Knock-down of eng in cultured human ECs does not affect cell alignment with the flow direction (Sugden et al., 2017) while, eng loss disrupts cell polarity evaluated by the relative Golgi and nuclei positions and causes migration defects in mice (Jin et al., 2017). Importantly, it was shown that *Eng* dependent induction of BMP 9/10 signaling is enhanced by flow (Baeyens et al., 2016b). Deletion of *Smad4* leads to an increase of the coronary artery caliber upon blood flow initiation, which is concurrent with activation of Smad1/5/8 (Poduri et al., 2017). The abnormal expansion of the coronary artery observed *in vivo* after depletion of *Smad4* appears to be due to an increase in cell proliferation and cell size (Poduri et al., 2017) resembling what was observed in the nascent arteries of zebrafish *alk1* mutant embryos (Corti et al., 2011). However, the *in vitro* observations suggest that the artery caliber defects could also arise due to cell migration defects against the flow direction (Poduri et al., 2017) as previously observed in the developing vascular network of the mouse yolk sac (Udan et al., 2013) and the zebrafish head (Rochon et al., 2016). Since flow forces are different in these settings, the variability in the cell behaviors may reflect different EC adaptation to the experienced flow regime. A key factor in the cell response to flow and AVMs is certainly Connexin (Cx) 37, which is deferentially regulated by shear stress and Smad1/5 signaling, and is downregulated in AVMs (Peacock et al., 2020). ECs appear to use different behaviors to adapt vessel size in response to flow depending on the developmental context. So far, most of our knowledge about vessel diameter regulation comes from work done on arteries and it is likely that vessel identity affects cell response to flow. It is clear for example that flow participates in reinforcing arterial vs. venous identity (Buschmann et al., 2010; Orsenigo et al., 2012). Yet arterial and venous identity is strongly dictated by genetic factors that are established before flow is observed (Su et al., 2018; Weijts et al., 2018; Geudens et al., 2019). This is certainly a general feature where flow profiles are enhancing and maintaining genetic programs established in early developmental stages.

## Endothelial to Hematopoietic Transition

**FIGURE 3:**
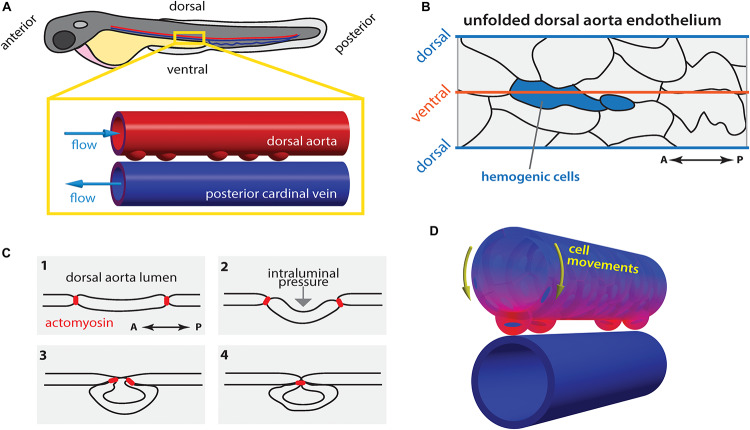
Hematopoietic stem cell emergence is blood flow dependent. **(A)** During definitive hematopoiesis, hematopoietic stem cell progenitors arise at the ventral side of the dorsal aorta (DA) via cell extrusion from the endothelium. **(B)** Potential hemogenic cells (blue) located at the ventral region of the DA are elongated along the anterior-posterior (AP) axis. **(C)** Cells are extruded from the endothelium by contraction of actomyosin that accumulated at the AP poles of the cell. **(D)** Concomitantly, the majority of endothelial cells move toward the ventral side of the DA (yellow arrows). At each heartbeat, the DA wall deforms asymmetrically: the deformation is highest (magenta) in the ventral region and lowest (blue) in the dorsal side. Drawings in **(B,C)** after Lancino et al. (2018).

Hematopoietic stem cell (HSC) emergence has been shown to occur *de novo* via endothelial-to-hematopoietic transition (EHT) from cells in the ventral wall of the DA (Figure 3A), so-called hemogenic endothelium, in both mouse and zebrafish embryos (Bertrand et al., 2010; Boisset et al., 2010; Kissa and Herbomel, 2010). During definitive hematopoiesis (Clements and Traver, 2013) emergent HSCs express the transcription factors runt-related transcription factor 1 (*Runx1*) and *cmyb*, which are amongst the earliest markers allowing HSC identification (Burns et al., 2002; Kalev-Zylinska et al., 2002; North et al., 2002). The ventral region of the DA is constantly exposed to blood flow and, in zebrafish at least, corresponds to an area that is highly deformable in response to pulsatile blood flow (Anton et al., 2013; Campinho et al., 2018). Indeed, work in cultured mouse embryonic stem cells (Adamo et al., 2009) as well as in zebrafish embryos (North et al., 2009; Lam et al., 2010) demonstrated that HSC differentiation is strongly dependent on blood flow-derived mechanical forces. It reflects the requirement for a sequential series of events involving the expression of the transcription factor *klf2a* (Wang et al., 2011) and the activation of nitric oxide synthase (North et al., 2009) in response to flow. Blocking primary cilia formation or function in the zebrafish embryo leads to defects in HSC specification (Liu et al., 2019) which is in agreement with a previously suggested cilia-dependent shear-sensing mechanism (Goetz et al., 2014). In the developing mouse embryo, shear stress acts through prostaglandin (PG) E2-cyclic adenosine monophosphate (cAMP)-protein kinase (PK) A, the cAMP response element-binding (CREB) protein and BMP signaling pathway to regulate HSC emergence via EHT (Diaz et al., 2015; Kim et al., 2015). In zebrafish, binding of adenosine to the A2b adenylyl cyclase-stimulatory receptor on the endothelium leads to up-regulation of the cAMP–PKA–CREB pathway to activate HSC development suggesting that this mechanism is conserved in vertebrates (Jing et al., 2015). In addition, Yap is involved in propagating flow force signals intracellularly and promotes the maintenance of HSC cell identity within the hemogenic endothelium (Lundin et al., 2020).

In zebrafish, the hemogenic cells undergo stereotypic shape changes during EHT leading up to cell extrusion from the endothelium in a process that requires actomyosin contractility and is hindered in the absence of blood flow (Figures 3B,C; Lancino et al., 2018). Hemogenic cells are elongated along the vessels length (anterior-posterior axis; Figure 3B) and actomyosin becomes enriched at the anterior-posterior poles of the cell, which are brought together by periodic contraction of the actomyosin network (Figure 3C; Lancino et al., 2018). Flow-forces possibly push the extruding cell outwards causing it to bulge out and acquire its, previously described (Kissa and Herbomel, 2010), characteristic cup-shaped morphology (Figure 3C).

While it is well established that flow-derived shear stress plays a crucial role in EHT and HSC development, it has only recently become clear that other flow-derived forces are involved in the process such as their cyclic component (Lundin et al., 2020) as initially suggested (Adamo et al., 2009). During the peak of EHT there is a generalized movement of ECs toward the ventral DA (extrusion region; yellow arrows Figure 3D; Poullet et al., 2019; Campinho et al., 2018), which corresponds to the region of greatest tissue deformation and computed tissue stress caused by pulsatile blood flow (Figure 3D; Campinho et al., 2018). Additionally, low blood flow leads to increased EC extrusion. As a consequence, the time spent by hemogenic cells in the ventral DA where they experience the appropriate biomechanical environment (shear and cyclic stretch) decreases when flow forces are too low (Campinho et al., 2018). At the cellular and tissue scale, the contribution of pressure-induced cyclic tissue-stretch to EHT and HSC development is thus to guide ECs to the ventral DA and regulate the extrusion rate. Yet, further experimental proof is necessary particularly if pressure-induced cyclic tissue-stretch is to be disentangled from the role of shear stress. *In vitro* approaches where shear and stretch can be applied in isolation will certainly help to address this issue.

## Conclusion

How blood flow forces affect vascular morphogenesis is an exciting field of research. Flow effects on cell polarization, shape changes and migration are obviously at the center of blood flows morphogenetic activity. Yet how these cell behaviors are coordinated with the angiogenic genetic programs, developmental pre-patterning and mechanotransduction is far from being understood. The mechanical environment that ECs experience is also highly relevant, since it affects the way cells react to forces (Ruehle et al., 2020). In addition, the contribution of different flow-derived forces (e.g., shear stress and circumferential stretch) to specific EC behaviors involved in vascular morphogenesis still needs to be clarified. These open questions are difficult and will benefit from a number of new tools: optogenetics (Chow and Vermot, 2017; Krueger et al., 2019), functionalized protein binders (Bieli et al., 2016), three dimensional live-imaging and processing (Campinho et al., 2020; Prahst et al., 2020) as well as tissue engineering approaches where forces are trackable and accurately controlled (Duchemin et al., 2019b; Vianello and Lutolf, 2019).

## Author Contributions

All the authors participated in the design and writing of the manuscript.

## Conflict of Interest

The authors declare that the research was conducted in the absence of any commercial or financial relationships that could be construed as a potential conflict of interest.
